# Drug-Related Felony Processing in Nevada (2016–2023): Trends in Arrest, Conviction, and Structural Exposure

**DOI:** 10.7759/cureus.105177

**Published:** 2026-03-13

**Authors:** Oscar Toro Ruilowa, Justin B Atkins, Marcella A Atkins, Anna L Stone

**Affiliations:** 1 Medicine, Kirk Kerkorian School of Medicine, University of Nevada, Las Vegas (UNLV), Las Vegas, USA; 2 Sociology, American Military University, Las Vegas, USA; 3 Public Defense, Clark County Public Defender's Office, Las Vegas, USA

**Keywords:** arrest trends, collateral consequences, criminal justice exposure, drug-related offenses, felony conviction, health policy, public health, social determinants, structural vulnerability, substance use disorders

## Abstract

Drug-related criminal justice involvement has increasingly been recognized as a structural factor influencing public health and socioeconomic outcomes, particularly among individuals with substance use disorders. While incarceration has been widely studied, less attention has been paid to felony-level criminal processing in cases where conviction or incarceration does not occur. This study was conducted as a narrative review and descriptive analysis of public health and criminal justice system data using publicly available administrative data from the Nevada Department of Health and Human Services (DHHS), Office of Analytics, to examine longitudinal patterns in drug-related arrests, drug-related convictions, and felony drug convictions in Nevada from 2016 through 2023.

From 2016 to 2023, drug-related arrests represented between 11.9% (5,403 of 45,247) and 19.6% (6,758 of 34,502) of arrests that ultimately resulted in conviction, with 3,129 of 16,049 (19.5%) reported in 2023. During the same period, drug-related convictions accounted for between 10.2% (3,775 of 37,107) and 15.2% (7,401 of 48,570) of total convictions, including 2,115 of 16,049 (13.2%) in 2023. Beginning in 2020, arrest proportions were higher than conviction proportions within adjudicated cases. The percentage of felonies attributable to drug-related offenses rose from 17.3% (1,385 of 7,991) in 2016 to 41.3% (896 of 2,170) in 2023. Similarly, the proportion of drug-related convictions classified as felonies increased from 19.2% in 2016 to 42.4% in 2023.

Among forensically involved individuals between fiscal years 2021 and 2023, 45.4% (816 of 1,798) self-reported drug use, and 34.2% (465 of 1,361) had a documented drug-related arrest. Together, these findings indicate sustained exposure to felony-level criminal processing within adjudicated populations without proportional increases in conviction severity or incarceration. When considered alongside established evidence documenting employment and earnings disadvantages associated with felony conviction status, these patterns underscore the importance of examining criminal justice system contact beyond incarceration metrics and highlight the structural implications of felony-level processing for long-term socioeconomic and health trajectories.

## Introduction

Criminal justice involvement is increasingly recognized as a determinant of health, with growing evidence that legal system exposure is associated with adverse physical, mental, and social outcomes. Mass incarceration and criminal legal contact have been linked to widening health disparities, chronic stress exposure, and long-term morbidity across affected populations [1-3]. Individuals with substance use disorders (SUDs) are disproportionately affected by criminalization policies, often encountering arrest, prosecution, and long-term legal consequences that extend beyond periods of incarceration [4,5]. Employment disadvantage and reduced labor market participation associated with felony history further compound downstream socioeconomic vulnerability and structural instability [6]. While incarceration itself has been extensively studied as a driver of health inequities [1,2], less attention has been paid to the health implications of felony-level criminal processing and other forms of criminal legal contact, particularly in cases where incarceration is limited or absent [7,8].

Drug-related offenses represent a substantial portion of criminal justice system contact in the United States, yet arrest does not uniformly result in conviction. Exposure to felony arrest and prosecution can nevertheless trigger durable collateral consequences, including barriers to employment, housing, healthcare access, and civic participation [4-6]. Civic exclusion and institutional disqualification further reinforce these disparities [9]. For individuals with SUDs, such structural barriers may undermine recovery, exacerbate mental health conditions, and perpetuate cycles of socioeconomic instability [1,4].

Nevada provides a useful case study for examining these dynamics. Over the past decade, the state has experienced shifts in drug policy, enforcement practices, and incarceration patterns while continuing to report substantial drug-related arrest activity. Publicly available administrative data from the Nevada Department of Health and Human Services (DHHS) demonstrate that drug-related arrests consistently exceed drug-related convictions, reflecting attrition across distinct procedural stages and suggesting sustained exposure to criminal processing without proportional adjudicated guilt [10]. In parallel, state-level data indicate a high prevalence of substance use among forensically arrested individuals, underscoring the intersection of substance use, criminal justice contact, and public health vulnerability [10].

Arrest, conviction, incarceration, and felony classification represent distinct procedural stages within the criminal legal system, each carrying different legal and social implications. This study focuses on arrests that ultimately resulted in conviction to examine the composition of adjudicated cases rather than the full population of arrests, which includes charges that may be dismissed or reduced. Felony classification is examined as a structural exposure because felony status can generate durable legal and socioeconomic consequences that extend beyond incarceration itself, including barriers to employment, housing, and civic participation. Clarifying these distinctions is essential for interpreting how criminal legal processes may influence long-term health and social outcomes.

Comparative data from the United States Sentencing Commission further contextualize Nevada’s experience within broader national incarceration and sentencing trends, illustrating that state-level arrest and conviction patterns operate within a complex correctional landscape that does not always align with federal sentencing patterns [11]. At the population level, criminal legal exposure also intersects with elevated morbidity and mortality risk among individuals cycling through incarceration and release, and with high-risk substance use patterns that carry significant public health consequences [12,13]. Together, these data support examining drug felony processing not solely as a legal outcome, but as a structural exposure with potential long-term health and socioeconomic consequences.

The objective of this study is to examine trends in drug-related arrests, convictions, and felony drug convictions in Nevada from 2016 to 2023 and to contextualize these trends within a public health framework and inform policy-relevant discussions regarding criminal justice system practices affecting individuals with substance use disorders. This study is motivated by the premise that exposure to felony-level criminal processing among individuals with substance use disorders may be associated with adverse long-term social and health-related outcomes independent of incarceration duration. By reframing drug felony processing as a structural health exposure rather than solely a legal outcome, this analysis aims to inform clinicians, public health professionals, and policymakers seeking to reduce preventable harm and strengthen recovery-oriented approaches to substance use disorders.

## Materials and methods

Study design

This study was conducted as a narrative review and descriptive public health and criminal justice systems analysis examining trends in drug-related arrests, convictions, and felony drug convictions in Nevada from 2016 through 2023. The analysis was designed to characterize patterns of criminal justice system exposure related to drug offenses using publicly available administrative data and to contextualize these patterns within established public health and sociological frameworks addressing structural disadvantage, structural competency, and collateral consequences of felony status [14].

Data sources

Data were obtained from publicly accessible reports published by the Nevada DHHS, Office of Analytics. These sources included statewide behavioral health datasets and the Substance Use and Criminality in Nevada analytic reports, which provide annual aggregate counts of arrests resulting in conviction, drug-related arrests, drug-related convictions, felony drug convictions, and substance use indicators among forensically arrested individuals [10]. Data were extracted directly from publicly available annual summary tables provided in the DHHS reports, and reported values were transcribed into structured datasets for descriptive analysis.

To contextualize state-level findings within broader sentencing and incarceration patterns, comparative federal sentencing data for Nevada were obtained from the United States Sentencing Commission’s 2023 state-level sentencing statistics [11]. Federal data were used solely for contextual comparison and were not merged with state-level arrest or conviction datasets.

Published sociological research examining employment barriers and earnings penalties associated with felony conviction status was reviewed to inform interpretation of the structural implications of felony-level criminal processing [4-6]. These sources were used for contextual interpretation rather than as primary datasets within the descriptive trend analysis.

All data sources were de-identified, aggregated, and publicly available. As a result, institutional review board approval was not required for this analysis.

Measures

Primary measures included annual totals and proportions of drug-related arrests, drug-related convictions, and felony drug convictions in Nevada from 2016 through 2023, as reported by the Nevada DHHS Office of Analytics [10]. Drug-related offenses and felony classifications were defined according to the standardized reporting categories used in Nevada DHHS administrative datasets, which classify offenses based on statutory charge codes and adjudication outcomes. Proportions were calculated by dividing drug-related arrests and convictions by the subset of arrests that ultimately resulted in conviction for each reporting year. This denominator reflects adjudicated cases rather than the full population of arrests. Because arrests and convictions represent distinct procedural stages, these proportions should not be interpreted as arrest-to-conviction conversion rates.

Secondary measures included reported substance use characteristics among forensically arrested individuals, as described in the Substance Use and Criminality in Nevada reports [10]. These measures were used to contextualize the intersection between substance use disorders and criminal justice system exposure rather than to establish individual-level associations.

Analysis

Descriptive statistical methods were used to examine longitudinal patterns in drug-related arrests, convictions, and felony drug convictions over the study period. All descriptive statistical analyses were conducted using IBM SPSS Statistics for Windows, Version 31 (Released 2025; IBM Corp., Armonk, New York, United States). Year-over-year comparisons and proportional analyses were conducted to describe differences between arrest exposure and adjudicated outcomes related to drug offenses within reported datasets. Because the study was designed as a descriptive analysis of administrative data, formal inferential statistical testing was not conducted, and observed differences should be interpreted as descriptive patterns rather than statistically validated trends.

Graphical visualizations were used to illustrate patterns in the proportion of arrests and convictions attributable to drug-related offenses over time. Findings were interpreted within established public health frameworks addressing social determinants of health and structural vulnerability [3,14], as well as sociological literature describing collateral consequences and stratification associated with felony conviction status [4-6,9,15].

Limitations of data sources

This analysis relies on administrative reporting, which may be subject to changes in enforcement practices, reporting definitions, and data collection methods over time. Additionally, the data do not permit individual-level linkage between arrest, conviction, incarceration duration, or health outcomes. The sociological literature cited to contextualize socioeconomic disparities reflects broader U.S. populations and may not directly quantify Nevada-specific outcomes. Accordingly, findings are descriptive and intended to inform hypothesis generation rather than causal inference. Year-specific variations in administrative reporting formats were addressed by applying consistent category definitions across reporting years based on DHHS documentation; however, minor inconsistencies in reporting structure may persist.

This analysis examines arrests that resulted in conviction rather than all recorded arrests. Consequently, the proportional measures describe the composition of adjudicated cases and do not estimate probabilities of arrest-to-conviction conversion or broader procedural attrition across criminal legal stages.

## Results

Drug-related arrests and convictions in Nevada (2016-2023)

From 2016 through 2023, drug-related arrests in Nevada consistently represented a minority of arrests that ultimately resulted in conviction. In 2016, 6,964 of 46,276 arrests (15.0%) were drug-related. This proportion declined to 5,403 of 45,247 (11.9%) in 2017 and then increased in subsequent years [10].

**Figure 1:**
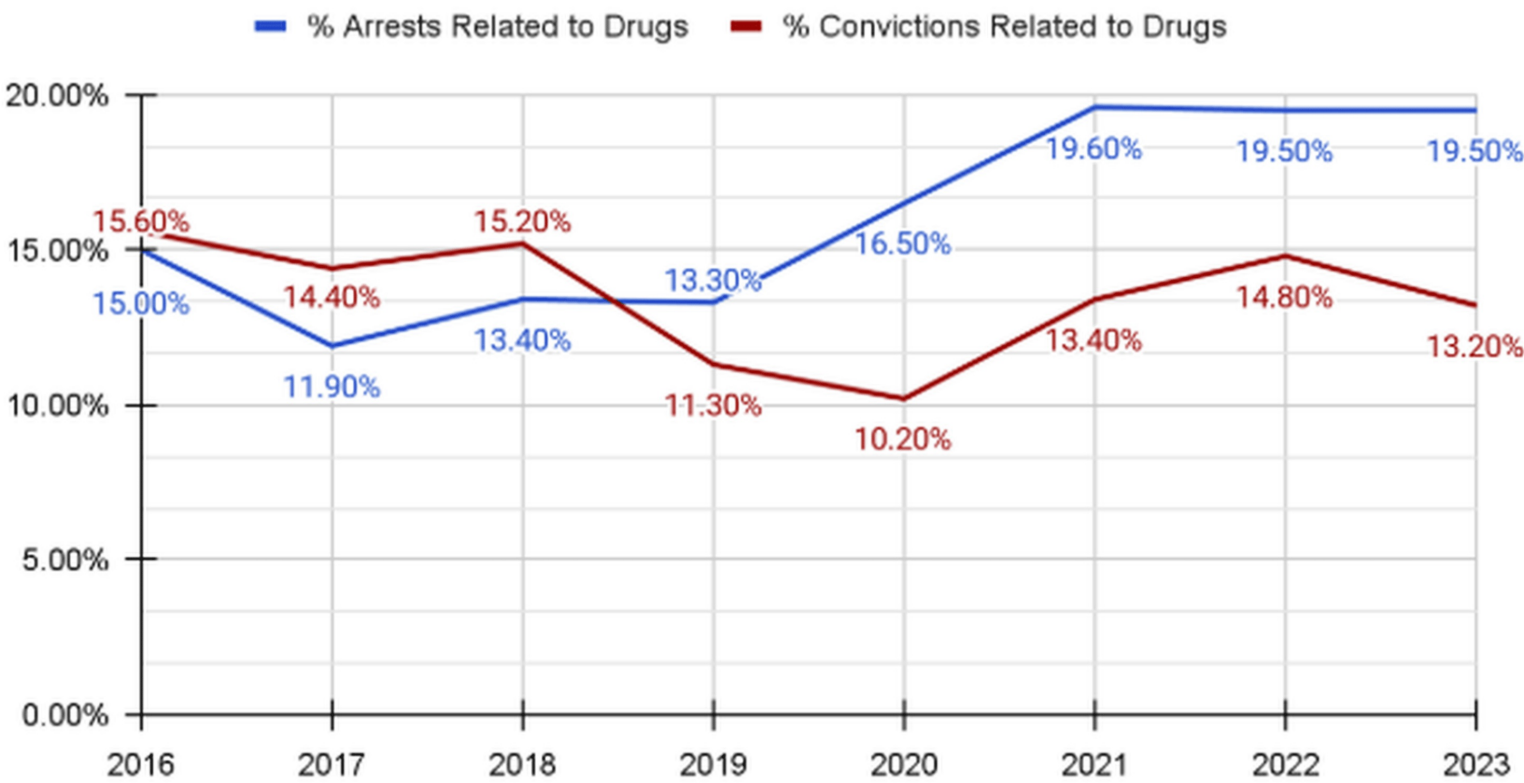
Substance-Related Arrests and Convictions in Nevada, 2016–2023. Percentage of arrests that ultimately resulted in conviction that were drug-related and percentage of total convictions that were drug-related in Nevada from 2016 through 2023. Data derived from publicly available Nevada Department of Health and Human Services reports [10].

Similarly, drug-related convictions accounted for a smaller proportion of total convictions during this period. Beginning in 2020, the proportion of arrests attributable to drug-related offenses was higher than the proportion of convictions, indicating a descriptive gap between arrest exposure and adjudicated outcomes over time. These patterns are presented in Figure 1.

Felony drug convictions over time

Felony drug convictions demonstrated variation across the study period. In 2016, 1,385 of 7,991 felony convictions (17.3%) were attributable to drug-related offenses. This proportion increased to 700 of 2,053 (34.1%) in 2018, declined to 299 of 1,839 (16.3%) in 2019, and then rose beginning in 2020 to 833 of 3,282 (25.4%). The proportion continued to increase to 2,440 of 5,935 (41.1%) in 2021 and reached its highest observed level at 2,380 of 5,532 (43.0%) in 2022 before remaining elevated at 896 of 2,170 (41.3%) in 2023 [10].

**Figure 2:**
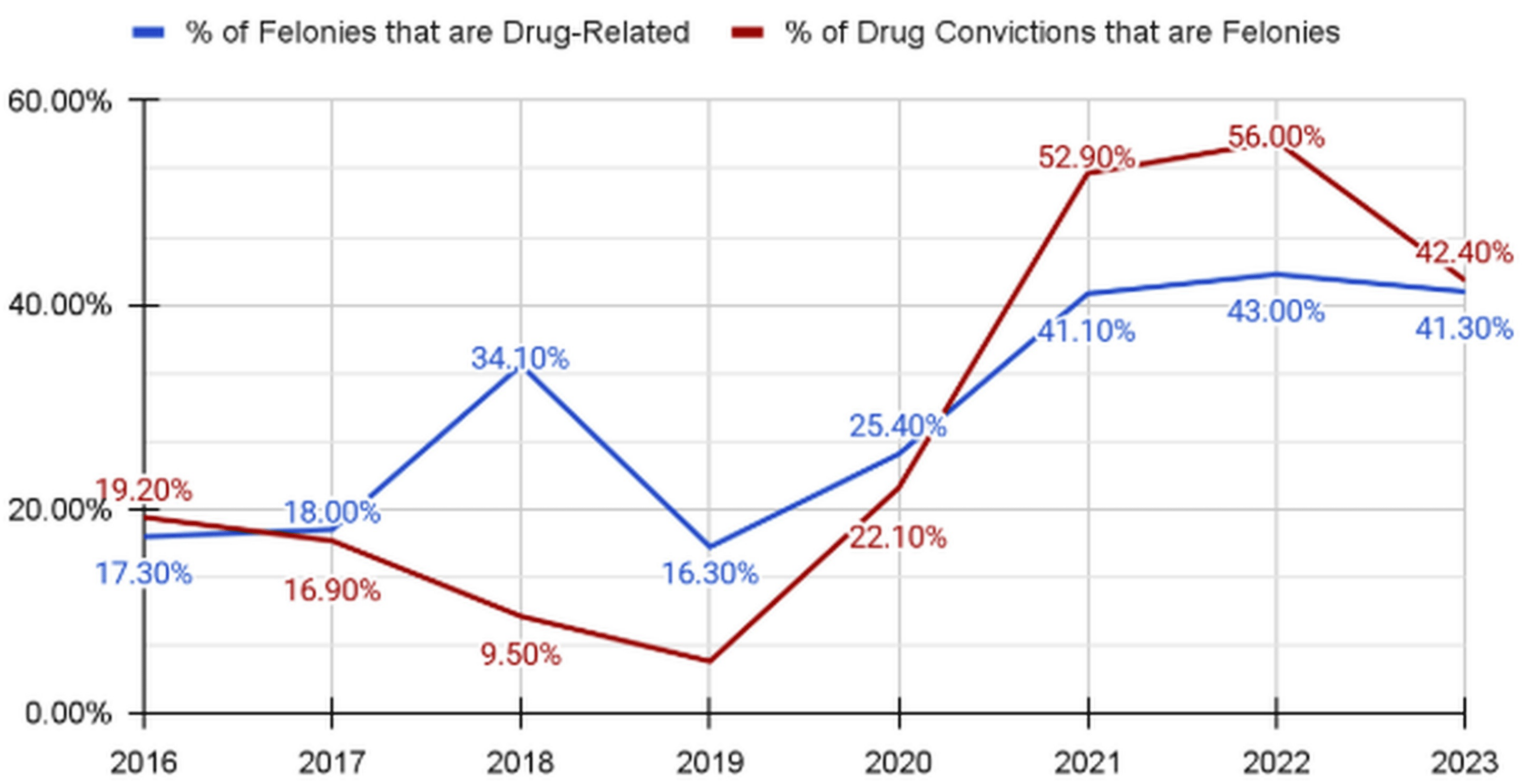
Felony Drug Convictions in Nevada, 2016–2023. Percentage of total felonies attributable to drug-related offenses and percentage of drug-related convictions classified as felonies in Nevada from 2016 through 2023. Figure created by the authors using publicly available Nevada Department of Health and Human Services data [10].

Similarly, the proportion of drug-related convictions classified as felonies increased after 2019. In 2018, 700 of 7,401 drug-related convictions (9.5%) were classified as felonies. This proportion declined to 299 of 5,839 (5.1%) in 2019 and then rose to 833 of 3,775 (22.1%) in 2020, 2,440 of 4,609 (52.9%) in 2021, and reached its highest observed level at 2,380 of 4,252 (56.0%) in 2022 before declining to 896 of 2,115 (42.4%) in 2023 [10]. These patterns are presented in Figure 2.

Felony status and socioeconomic indicators

**Figure 3:**
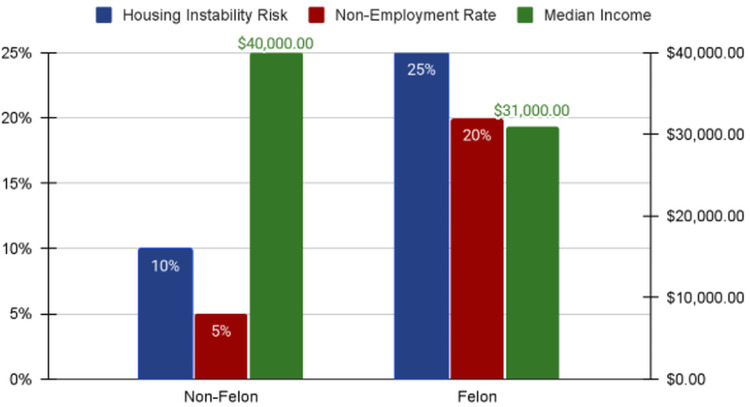
Felony Status and Socioeconomic Indicators. Comparative labor force participation, non-employment rates, and median annual income among individuals with and without felony conviction status, derived from published U.S. sociological research [4–6,15].

Published sociological research has documented persistent socioeconomic disparities associated with felony conviction status, including reduced labor force participation, elevated non-employment rates, and long-term earnings differences relative to individuals without felony records [4-6,15]. Prior analyses have demonstrated that individuals with felony convictions experience higher rates of housing instability, higher non-employment rates, and lower median annual income relative to individuals without felony records [4-6,15]. These comparative indicators are displayed in Figure 3 to contextualize the structural implications of felony-level criminal processing.

Collateral consequences associated with felony status

In addition to measurable socioeconomic disparities, felony conviction status is associated with structured limitations across multiple civic and institutional domains. Individuals with felony convictions may face restrictions related to public housing eligibility, Supplemental Nutrition Assistance Program (SNAP) benefits, student loan eligibility, employment disclosure requirements, voting rights, jury service eligibility, professional licensure, and access to certain federally subsidized housing programs [4,5,9,15]. While the specific scope and duration of these restrictions vary by jurisdiction and offense type, felony conviction status frequently functions as a formal or informal barrier across these domains [4,5,9,15]. These domain-level differences are summarized in Table 1.

**Table 1:** Selected Collateral Consequences Associated with Felony Conviction Status. Comparison of selected civic, employment, housing, and benefits-related domains associated with felony conviction status. Policies vary by jurisdiction and offense type. Table created by the authors based on published legal and policy sources [4,5,9,15].

Domain	No Felony Conviction	Felony Conviction
Public Housing	Generally eligible	May be restricted or denied depending on offense and jurisdiction
SNAP Benefits	Generally eligible	May be restricted depending on offense and state policy
Student Loans	Eligible	May be limited depending on offense and applicable federal policy
Employment	No mandatory disclosure of felony status	Disclosure of felony conviction may be required; may affect hiring
Voting	Eligible	May be restricted depending on jurisdiction
Jury Service	Eligible	May be disqualified depending on jurisdiction
Professional Licensure	Eligible	May be limited depending on offense and regulatory body
Federally Subsidized Housing	Eligible	May be excluded depending on offense history

Summary of results

Across the study period, drug-related arrests consistently represented a higher proportion than drug-related convictions when calculated relative to the total number of arrests that ultimately resulted in conviction. For example, in 2023, 3,129 of 16,049 arrests that resulted in conviction (19.5%) were drug-related, compared with 2,115 of 16,049 total convictions (13.2%). Similarly, in 2021, 6,758 of 34,502 arrests that resulted in conviction (19.6%) were drug-related, compared with 4,609 of 34,502 total convictions (13.4%) [10].

Felony classification among drug-related offenses increased beginning in 2020, with drug-related felonies rising from 299 of 1,839 total felonies (16.3%) in 2019 to 2,380 of 5,532 (43.0%) in 2022 before declining to 896 of 2,170 (41.3%) in 2023 [10].

In parallel, published sociological research demonstrates that felony conviction status is associated with measurable socioeconomic disparities and structured collateral consequences across multiple domains [4-6,9,15]. Together, these findings describe sustained patterns of felony-level criminal processing within a broader structural context.

## Discussion

This analysis describes a consistent difference between drug-related arrests and drug-related convictions in Nevada from 2016 to 2023, with drug-related arrests consistently exceeding convictions as a proportion of total arrests resulting in conviction. For example, in 2021, 6,758 of 34,502 arrests (19.6%) were drug-related compared with 4,609 of 34,502 convictions (13.4%), and a similar descriptive gap remained evident in 2023 (19.5% vs 13.2%) [10]. Despite fluctuations in overall arrest activity across the study period, including during the COVID-19 pandemic, felony drug convictions did not increase proportionally, a pattern consistent with sustained exposure to felony-level criminal processing without corresponding increases in adjudicated outcomes or incarceration.

These findings are notable because they highlight criminal justice system exposure as a potential source of structural vulnerability independent of conviction or incarceration. While incarceration has been widely studied as a determinant of adverse health outcomes and widening inequality [1,2], the present analysis underscores that felony arrest and prosecution alone may function as meaningful structural exposures [3,14]. Even in the absence of conviction, individuals may experience durable collateral consequences that affect employment opportunities, housing stability, access to healthcare, and engagement with social services [4-6,15]. Sociological research has described felony conviction status as a durable social marker shaping labor market participation and long-term earnings trajectories through mechanisms of exclusion and stigma [4-6]. Within this framework, felony processing operates not merely as a legal classification but as a stratifying mechanism influencing long-term social position [3,14].

The high prevalence of reported substance use among forensically arrested individuals further contextualizes these findings. Between fiscal years 2021 and 2023, 45.4% of forensic patients self-reported drug use, and 34.2% had a documented drug-related arrest [10]. This overlap between substance use disorders and criminal justice involvement is consistent with prior epidemiologic research documenting the intersection of substance use, incarceration, and elevated health risk [12,13]. When legal system exposure interacts with underlying socioeconomic disadvantage, cumulative effects may reinforce instability and limit access to treatment and recovery resources [1,3]. From both public health and sociological perspectives, this interaction reflects how institutional processes can amplify pre-existing vulnerabilities [3,14].

Importantly, the increasing proportion of drug-related convictions classified as felonies after 2020, reaching its highest observed level at 56.0% in 2022, occurred alongside a persistent descriptive gap between arrest exposure and conviction proportions [10]. This pattern warrants further examination regarding the structural and health implications of enforcement-focused approaches. The difference between arrest exposure and adjudicated outcomes indicates that many individuals experience the burdens of felony processing without a clear legal resolution. Sociological literature on collateral consequences indicates that felony conviction status can produce lasting effects on employment prospects, labor force participation, and earnings potential even beyond periods of incarceration [4-6,15]. From a public health standpoint, such structural barriers may contribute to chronic stress and reduced access to preventive care [1,2].

Nevada’s experience also reflects broader national dynamics. Comparative federal sentencing data indicate that state-level arrest and conviction patterns operate within a complex correctional landscape in which incarceration rates, sentencing practices, and enforcement priorities do not always align [11]. This reinforces the importance of examining criminal justice exposure beyond incarceration metrics alone. A focus solely on imprisonment may underestimate the health and social consequences of earlier points of system contact, including arrest and prosecution, which prior research suggests can independently shape institutional trust, system avoidance, and socioeconomic outcomes [7].

Taken together, these findings support conceptualizing felony drug processing as a structural exposure rather than a discrete legal event. Similar to other non-reversible exposures that alter social position, felony processing may constrain access to employment, housing, and civic participation in ways that extend beyond formal sentencing [3,14]. Recognizing felony-level criminal processing as both a public health and sociological phenomenon may help inform approaches that prioritize treatment engagement, recovery support, and mitigation of collateral consequences while maintaining public safety objectives. These contextual socioeconomic findings derive from national sociological studies and are presented to inform interpretation rather than to represent Nevada-specific measurements.

Limitations

This study has several important limitations. First, the analysis relies on aggregated administrative data derived from publicly available state and federal sources. As a result, individual-level linkage between arrests, convictions, incarceration duration, substance use diagnoses, healthcare utilization, or downstream health outcomes was not possible. The findings therefore describe population-level patterns rather than individual trajectories. Additionally, the administrative data capture aggregate arrest and conviction events rather than the full procedural experiences of individuals; therefore, references to “felony-level criminal processing” should be interpreted as system-level patterns rather than direct measurement of individual exposure.

Second, variation in enforcement practices, charging decisions, reporting definitions, and data collection methods over time may influence observed trends. Changes in policing priorities, court operations, and administrative processes, particularly during the COVID-19 pandemic, could contribute to year-to-year fluctuations not fully captured in the available data.

Third, the descriptive design of this study precludes causal inference. Although the observed difference between drug-related arrests and convictions suggests sustained exposure to felony-level criminal processing without proportional adjudicated outcomes, the analysis cannot determine direct health effects, establish causal pathways, or isolate the impact of specific policy or enforcement changes. Additionally, inferential statistical testing was not performed; therefore, observed differences and longitudinal patterns should be interpreted as descriptive rather than statistically validated trends. Although this study informs policy-relevant discussions by describing statewide trends in criminal justice system exposure, it does not directly analyze specific legislative changes, enforcement policies, or implementation of formal policy interventions.

Fourth, analyses were based on administrative counts and proportions rather than population-standardized rates. Consequently, temporal changes may partially reflect underlying demographic shifts, population growth, or changes in age distribution rather than solely changes in enforcement patterns or legal processing dynamics. Because publicly available administrative datasets did not provide linked annual population denominators across reporting categories, rate standardization was not feasible. Findings should therefore be interpreted as descriptive of system-level patterns rather than population-level incidence.

Fifth, socioeconomic disparities associated with felony status were contextualized using published sociological research rather than Nevada-specific individual-level data. While prior studies have documented employment disadvantages and earnings penalties associated with felony conviction status in U.S. populations [4-6,15], the present study did not independently measure these outcomes within the Nevada dataset.

Finally, this analysis focuses on a single state. While Nevada provides a useful case study and reflects broader national trends, findings may not be generalizable to states with different legal frameworks, enforcement practices, or healthcare infrastructures.

Taken together, these findings support conceptualizing felony drug processing as a structural exposure rather than a discrete legal event. Similar to other non-reversible exposures that alter social position, felony processing may constrain access to employment, housing, and civic participation in ways that extend beyond formal sentencing [3,14]. Recognizing felony-level criminal processing as both a public health and sociological phenomenon may help inform approaches that prioritize treatment engagement, recovery support, and mitigation of collateral consequences while maintaining public safety objectives. These findings provide policy-relevant insight into how criminal justice system practices may generate sustained structural exposure for individuals with substance use disorders, even in the absence of incarceration.

## Conclusions

This analysis demonstrates a persistent difference between drug-related arrests and convictions in Nevada from 2016 to 2023, with arrests consistently exceeding adjudicated outcomes. Despite fluctuations in overall arrest activity, including during periods of system disruption, felony drug convictions did not increase commensurately with arrest exposure. These findings are consistent with sustained system-level patterns of felony-level criminal processing for drug-related offenses without corresponding increases in conviction or incarceration, suggesting potential structural implications beyond formal sentencing outcomes.

From a public health and sociological perspective, this pattern underscores the importance of examining criminal justice system contact beyond incarceration metrics alone. Felony arrest and prosecution may function not only as legal events but also as structural exposures that shape access to employment, housing, and economic mobility. When considered alongside established evidence documenting the collateral socioeconomic consequences of felony status, these trends suggest that early points of criminal justice contact may carry potential long-term implications for social position and health, particularly among individuals with substance use disorders. Future research should prioritize individual-level analyses to clarify long-term health, social, and economic outcomes and to inform balanced approaches that promote recovery, stability, and public safety.
